# Reduced pharyngeal pumping rates observed in *tph-1* mutants using microfluidic electropharyngeogram (EPG) recordings

**DOI:** 10.17912/W2CC7Z

**Published:** 2017-02-08

**Authors:** Terra Hiebert, Adela Chicas-Cruz, Kathryn McCormick

**Affiliations:** 1 NemaMetrix, Inc,. 44 W 7th Ave., Eugene, OR 97401 USA.

**Figure 1.**
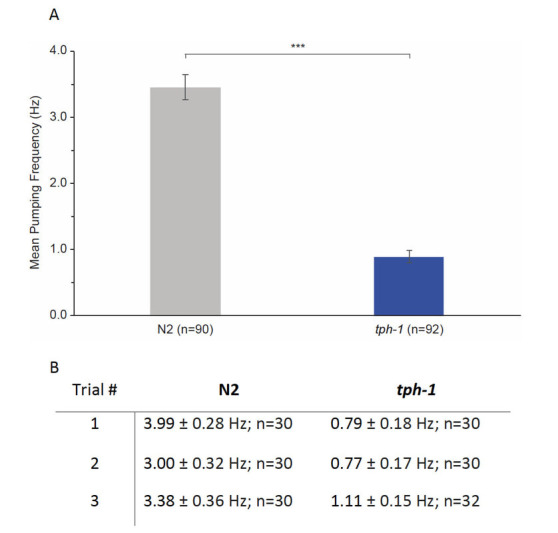


## Description

In *Caenorhabditis* *elegans,* serotonin (5-HT) activates and controls pharyngeal pumping in response to food (Horvitz et al., 1982; Sze et al., 2000; Song and Avery 2012). Tryptophan hydroxylase, the enzyme required for serotonin biosynthesis, is encoded by the gene *tph-1*. Worms with a *tph-1* deletion mutation exhibit phenotypes associated with a lack of serotonin-signaling, including reduced pharyngeal pumping (Sze et al., 2000; Avery and Horvitz 1990; Song and Avery 2012). We used a microfluidic electropharyngeogram (EPG) recording platform (NemaMetrix) and associated software (NemAnalysis) to measure pharyngeal pumping in *C. elegans* *tph-1* mutants in the presence of bacterial food (100 mg/ml *E. coli* OP50 in M9 buffer), following a 2-hr fasting period. Prior research has shown that a fasting period (e.g., 2-4-hr) induces elevated feeding rates for worms upon re-introduction to bacterial food (Lemieux and Ashrafi 2015). We chose to measure pharyngeal pumping during this elevated feeding phase due to our hypothesis that *tph-1* animals would exhibit lower pumping rates than control worms. Pumping was recorded for 2-minute durations over three independent trials (total N2 n = 90; *tph-1* n = 92). *C. elegans* *tph-1* mutants exhibited significantly lower pharyngeal pumping rates than N2 control animals (A, N2 = 3.46 ± 0.19 Hz; *tph-1* = 0.89 ± 0.10 Hz; mean ± SEM; *** p < 0.0001, 2-tailed students t-test). Pumping frequency data were pooled in A; see B for a comparison of each experimental trial.

## Reagents

Strains: MT15434 *tph-1* *(**mg280**)* II, kindly provided by the Prahlad lab.

Control Strain: N2
